# Correction: Early nasal type I IFN immunity against SARS-CoV-2 is compromised in patients with autoantibodies against type I IFNs

**DOI:** 10.1084/jem.2021121108132021c

**Published:** 2021-08-20

**Authors:** Jonathan Lopez, Marine Mommert, William Mouton, Andrés Pizzorno, Karen Brengel-Pesce, Mehdi Mezidi, Marine Villard, Bruno Lina, Jean-Christophe Richard, Jean-Baptiste Fassier, Valérie Cheynet, Blandine Padey, Victoria Duliere, Thomas Julien, Stéphane Paul, Paul Bastard, Alexandre Belot, Antonin Bal, Jean-Laurent Casanova, Manuel Rosa-Calatrava, Florence Morfin, Thierry Walzer, Sophie Trouillet-Assant

Vol. 218, No. 10 | 10.1084/jem.20211211 | August 6, 2021

The authors regret that in the original version of this paper, Hadjadj et al. (2020) was cited instead of Bastard et ala. (2020) in a sentence about auto-Abs in the Introduction. The corrected sentence is below.

“This defect is explained in some patients by inborn genetic defects of IFN-I induction or amplification (Zhang et al., 2020b) and in others by the presence of neutralizing autoantibodies (auto-Abs) against type I IFNs, mainly IFN-α and/or IFN-ω and, in rare cases, IFN-β (Bastard et al., 2020).”

The error appears only in PDFs downloaded before August 10, 2021.

